# Isolated Cardiac Sarcoidosis with High-Grade Heart Block: Utilization of New Diagnostic Guidelines

**DOI:** 10.1155/2021/9992678

**Published:** 2021-07-28

**Authors:** Matthew R. Petersen, Christopher Perry, Rachel Nickels

**Affiliations:** ^1^Department of Internal Medicine, University of Florida, USA; ^2^Department of Cardiology, University of Florida, USA; ^3^United States Department of Veterans Affairs, North Florida South Georgia VA Hospital, USA

## Abstract

Cardiac sarcoidosis can present with heart failure and conduction disease. This is a case of a 58-year-old male who presented for dyspnea, edema, and varying degrees of heart block. Using new updated diagnostic guidelines and multimodal cardiac imaging, he was diagnosed with isolated cardiac sarcoidosis.

## 1. History of Present Illness

A 58-year-old male presented to the emergency room with bradycardia and lower extremity edema. A few weeks prior, he originally presented to a neighboring hospital for new onset dyspnea on exertion and lower extremity edema. On arrival, ECG showed baseline atrial fibrillation with junctional escape rhythm at a rate of 35-40 beats per minute. He was normotensive, and his only complaints were lower extremity edema and subacute fatigue.

Physical exam revealed an obese middle-aged male in no acute distress. His was mildly tachypneic, was sitting upright on the edge of the bed, and his lung exam revealed bibasilar crackles. His heart rate was slowed but was in a regular rhythm. He had 2+ bilateral lower extremity edema, and his extremities were warm. Initial labs revealed hemoglobin of 13.4 g/dL, hematocrit 42.2%, and platelets 143 k/cmm with normal differential. Metabolic panel revealed electrolytes within normal limits; serum creatinine was 1.4 mg/dL which was slightly above the patients baseline of 1.2 mg/dL. Albumin, calcium, transaminases, and bilirubin were within normal limits. High sensitivity troponin was 32 ng/L with no change on serial repeat tests, and NT pro BNP was 3018 pg/mL.

## 2. Past Medical History

The patient had a past medical history of obesity and asymptomatic bradycardia first reported several years prior to presentation. He recently had been diagnosed with heart failure with preserved ejection fraction (EF) a few weeks prior at a neighboring hospital.

### 2.1. Differential Diagnosis

Due to the presentation of a middle age, normotensive black male with heart failure and bradycardia, there was suspicion for an infiltrative cardiomyopathy such as amyloidosis, both light chain and transthyretin types, as well as sarcoidosis with cardiac involvement.

### 2.2. Investigations

**Figure 1:**
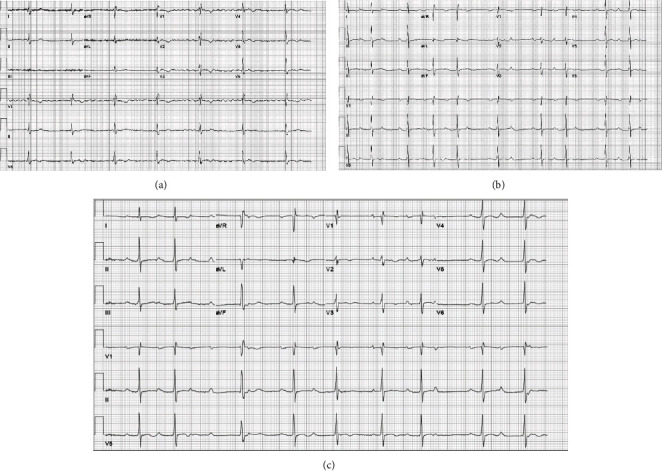
ECG of variable heart block in a patient with cardiac sarcoidosis: (a) baseline atrial fibrillation with junctional escape rhythm; (b) second-degree Mobitz type 1 AV block; (c) high grade AV block.

Serial ECGs showed several different bradycardic rhythms (Figure 1). He was noted to have intermittent second-degree Mobitz type 1 atrioventricular block (AVB) but also periods of high-grade AVB. Transthoracic echocardiogram revealed asymmetric left ventricular hypertrophy, normal systolic function (EF estimated at 55%), normal wall motion, and grade 1 diastolic dysfunction. There was no evidence of outflow tract obstruction, and ventricular filling pressures were estimated to be normal. He underwent a treadmill stress test, demonstrating chronotropic competency without evidence of ischemia. Cardiac magnetic resonance (CMR) imaging revealed asymmetric hypertrophy of the ventricular septum with patchy myocardial late gadolinium enhancement involving the mid- to basal septum with heterogeneous uptake in the apex. Labs revealed a normal protein electrophoresis making light chain amyloidosis unlikely. The patient underwent a CT scan of the chest to examine for evidence of pulmonary sarcoidosis; however, this was unremarkable. A technetium-pyrophosphate scan was obtained for assessment of transthyretin amyloid; however, this was negative as well.

**Figure 2:**
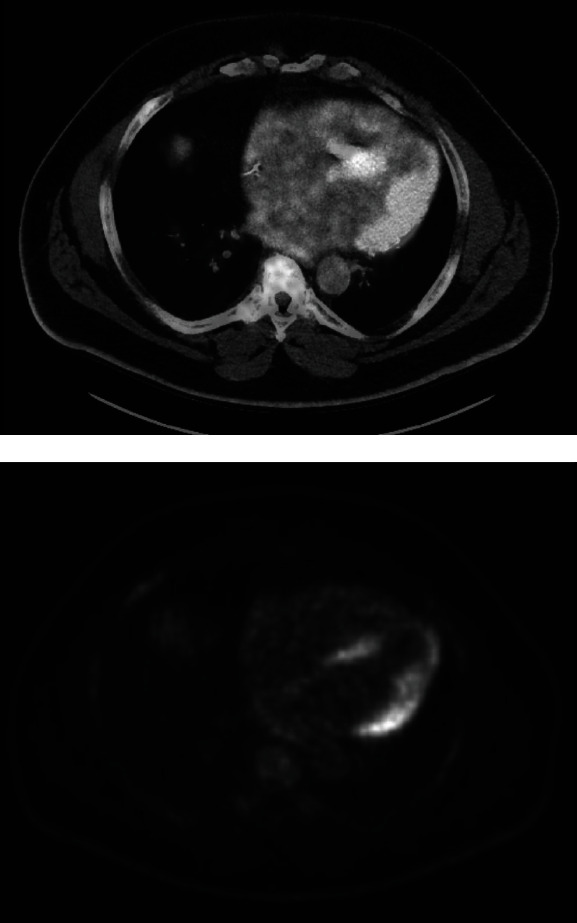
Active sarcoid seen in myocardium on FDG PET CT. FDG PET CT showing abnormal uptake in the myocardium as well as apical inferior wall with sparing of the apex with no extracardiac FDR uptake.

The patient was scheduled for follow-up Fluorodeoxyglucose Positron Emission Tomography-Computed Tomography (FDG PET CT) scan. When he obtained the scan, it was positive for FDG uptake in the left ventricular myocardium as well as the apical inferior wall, with no abnormal extracardiac FDG uptake (see Figure 2). These findings were consistent with active isolated cardiac sarcoidosis primarily involving the mid- to basal myocardium.

### 2.3. Management

During his hospitalization, the patient was administered intravenous loop diuretics for his volume overload. He underwent dual chamber pacemaker implantation, as well as empiric implantable cardiac defibrillator (ICD) placement for primary prevention against ventricular arrhythmias. ICD placement was felt appropriate despite not yet obtaining a definitive diagnosis of cardiac sarcoidosis (CS) for two reasons: (1) the patient's existing need for a pacemaker for treatment of bradycardia and high-grade AVB and (2) the high suspicion for CS. The team did not want to delay primary prevention therapy due to risk of sudden cardiac death in patients with CS and heart block. The patient's cardiac device was set to DDD (dual chamber paced, dual sensed, and dual inhibited) settings given his high degree of block as well as underlying paroxysmal atrial fibrillation. Upon follow-up interrogation of the device, the patient was paced from the right atrial lead 54% of the time and from the right ventricular lead 100% of the time and was in atrial fibrillation 28% of the time, showing his high-grade heart block and underlying bradycardia. He has not had any ventricular arrhythmias to date.

## 3. Discussion

Sarcoidosis is a granulomatous disorder of unknown etiology that can affect multiple organ systems. The histopathologic hallmark of sarcoidosis is the presence of noncaseating granulomas in affected organs. An estimated 5-25% of patients with sarcoidosis have cardiac involvement [1]. CS can cause a variety of cardiac problems, including heart failure and conduction disease. In a European registry of sarcoidosis patients, high-grade atrioventricular blocks were the most common presentation of those affected, seen in about 44% of CS patients [2]. Those with CS have increased risk of arrhythmias, particularly ventricular arrhythmias. One cohort study found that the 5-year incidence of VT was 56% in those with EF below 25% and 25% in those with normal EF [2]. There is also an increased risk of sudden cardiac death in CS patients ranging from 9% in those with normal EF and no history of VT to 34% in those with EF below 25% and prior history of VT [2]. Given these risks, diagnosing cardiac sarcoidosis early is imperative.

Previous guidelines for diagnosis required biopsy-proven disease either of the cardiac tissue or extracardiac tissue with other signs of cardiac involvement [3]. These and other European and American diagnostic guidelines have been too reliant on biopsy data, which has poor diagnostic yields, [4] and does not take into account recent advancements in cardiac imaging. New Japanese diagnostic guidelines for CS and isolated cardiac sarcoidosis (ICS) were published in 2017. These new guidelines proposed the diagnosis of CS via major and minor criteria to classify cardiac involvement that includes analysis of ECG, ECHO, CMR, and FDG PET findings (Table 1) [5]. These guidelines have been shown to capture more patients with CS and ICS, allowing them to proceed to recommended therapies such as steroid treatment and cardiac devices for primary prevention [6].

This patient was diagnosed with ICS based on these guidelines as he had FDG-PET data consistent with cardiac involvement without extracardiac involvement, high-grade AVB, abnormal ventricular wall anatomy, and late gadolinium enhancement seen on CMR.

Treatment with immunosuppression has little high-level data, and most recommendations are driven by expert consensus. One review that discusses induction of steroids for improvement of both ejection fraction and conduction disease described a modest improvement in conduction disease in those treated with steroids, as well as improvement in ejection fraction in those with initial EF below 35%, however no significant improvement in those with moderately reduced EFs [7]. One systematic review examining the benefits of steroid treatments in CS patients concluded that treatment with exogenous corticosteroids improved conduction disease in about 50% of those treated; however, there was too little data to support recommendations in steroids to treat ventricular arrhythmia burden or mortality [8].

### 3.1. Follow-Up

The patient followed up with outpatient cardiology after discharge. He continues on diuretics and rivaroxaban for anticoagulation given his atrial fibrillation. Follow-up ECHO 6 months after initial diagnosis showed reduced ejection fraction at 25%, and the patient was started on guideline-directed medical therapy for heart failure with reduced ejection fraction with betablocker, RAAS inhibition, and Aldactone. He underwent eye exam and thorough pulmonology workup, which was again negative for extracardiac manifestations of sarcoidosis, and the patient just recently was initiated on systemic corticosteroids at 40 mg daily for three months, with tapering dose to 5-15 mg daily thereafter. A repeat echocardiogram will be obtained in 3 months, and repeat cardiac device interrogation to examine for progression or improvement on conduction disease will occur at one-year follow-up.

## 4. Conclusion

This case of suspected CS with variable high-grade conduction blocks highlights the utilization of recently updated diagnostic guidelines for CS. This case illustrates the need to continue to develop progressive diagnostic guidelines in line with available technology. This can lead to more accurate and earlier diagnosis, which allows for more CS patients to benefit from cardiac device and steroid treatment for arrhythmias and primary prevention of sudden cardiac death.

## Figures and Tables

**(a):** 

	Cardiac sarcoidosis	Isolated cardiac sarcoidosis
Histological diagnosis group	(i) Endomyocardial biopsy or surgical specimens confirm noncaseating granulomatous tissue	(i) Prerequisite required^+^ (ii) Endomyocardial biopsy or surgical specimens confirm noncaseating granulomatous tissue
Clinical diagnosis group	(i) Patient diagnosed with sarcoidosis via tissue biopsy diagnosis of noncardiac site and (ii) Signs of cardiac involvement^∗^	(i) Prerequisite required^+^ (ii) F-FDG PET reveals abnormally high accumulation of radiotracer in cardiac tissue and (iii) 3/4 of the other major criteria for cardiac involvement^∗^ are met

**(b):** 

∗Cardiac involvement Requires 2/5 major criteria or 1/5 major criteria and 2/3 minor criteria	(i) Major criteria (a) High-grade AV block (b) Abnormal ventricular wall anatomy (c) LVEF < 50% (d) F-FDG PET with increased tracer uptake in cardiac tissue (e) Cardiac MRI with delayed gadolinium enhancement in myocardium (ii) Minor criteria (a) Abnormal ECG findings (b) Perfusion defects on myocardial perfusion scintigraphy (c) Cardiac biopsy with monocyte infiltration or fibrosis
^+^Prerequisite required for ICS	(i) No clinical findings of sarcoidosis in extracardiac organs and no signs of hilar or mediastinal lymphadenopathy (ii) F-FDG PET with no uptake accumulation in extracardiac organs

## Data Availability

There is no underlying data to report.

## Additional Points

*Learning Objectives*. (i) Cardiac sarcoidosis can present as heart failure or conduction disease such as high-degree AVB or ventricular arrhythmias. (ii) New Japan diagnostic guidelines allows for use of advanced cardiac imaging for diagnosis of CS and ICS, addressing the problems of poor diagnostic yield with endomyocardial biopsies and taking advantage of new medical technologies. (iii) Patients with CS, particularly those with reduced EF, are at high risk for ventricular arrhythmias and sudden cardiac death. These can be treated with ICD placement and systemic corticosteroids.
